# The prevalence of suicidal behaviour and its associated risk factors among school-going adolescents resident in the United Arab Emirates

**DOI:** 10.1038/s41598-023-47305-3

**Published:** 2023-11-15

**Authors:** Zahir Vally, Mai Helmy

**Affiliations:** 1 Department of Clinical Psychology, United Arab Emirates University, Al Ain, P. O. Box 15551, Abu Dhabi, United Arab Emirates; 2 Department of Psychology, Sultan Qaboos University, Al-Khod, Sultanate of Oman; 3 Psychology Department, Faculty of Arts, Menoufia University, Shebin El-Kom, Egypt

**Keywords:** Psychology, Risk factors

## Abstract

Suicidal behaviour which includes suicidal ideation, having a plan to commit suicide and suicide attempts remains a global public health issue as it substantially impacts adolescent health and wellbeing. Suicidal behaviour, however, remains understudied in Middle Eastern contexts. This study analysed data from the 2016 Global School-based Student Health Survey collected in the United Arab Emirates (UAE). A sample of 5826 adolescents aged between 11 and 18 years were sampled. Potential risk factors associated with an elevated risk for engagement in suicidal behaviour were examined. These factors were stratified into categories for analysis (demographics, psychosocial, risky health, and socio-environmental). The age-adjusted prevalence of suicidal behaviour amongst the school-going adolescent population in the UAE was 54%. Analyses indicated that elevated risk was significantly associated with anxiety difficulties, the experience of loneliness, and amongst those who smoked tobacco. None of the socio-environmental factors emerged as significant. A dose-dependent relationship was evident in that the degree of risk that was evident appeared to compound as the number of adverse risk factors increased. The data suggest that suicidal behaviour may be highly prevalent in this location. Findings highlight the immense need to develop preventative interventions, some of which may be school-delivered and targeted at parents. Our findings provide initial indications as to which risk factors could be targeted for remediation in developing these interventions.

## Introduction

Suicide continues to be regarded as a substantial global health issue which carries a substantial psychological impact that affects the afflicted individuals and their families but also transcends the individual level to impact the larger family and community at economic and social levels^1–4^. The prevention of suicide has been identified as an important target for organizations tasked with the promotion of large-scale public mental health campaigns; for example, the World Health Organization’s (WHO) Mental Health Action Plan has set itself the goal of reducing the incidence of suicide by at least 10% over an 8-year period^5^.

Whilst suicide may occur at any stage of the developmental spectrum, the adolescent years appear to be a particularly vulnerable stage of life for the occurrence of suicide. The WHO^6^ reports that suicide as well as the accidental deaths that may occur as a result of self-harming behaviours result in approximately 67,000 worldwide deaths each year, a figure that represents the second leading cause of mortality amongst the adolescent population. While suicide appears to occur disproportionately among adolescents in low and middle-income countries, three out of four suicides worldwide are reported to occur in these regions of the world^5^, research on suicidal behaviour has tended to focus almost exclusively on samples resident in high-income countries and amongst Caucasian populations, particularly those from Europe and North America. Moreover, the incidence of suicide appears to be more pronounced amongst particular subcategories of adolescents; adolescent girls, for example, as well as those resident in Southeast Asia appear to be more vulnerable where suicide is the leading cause of mortality^1,7,8^.

Epidemiological data suggests that the prevalence rates for adolescent suicidal behaviour vary widely between countries. Moreover, non-fatal suicidal behaviour (ideation with or without a plan and non-fatal attempts) appears to be equally problematic and, in some cases, more prevalent than fatal suicide. Multi-country studies provide a valuable overview of the range in prevalence rates. McKinnon et al.^9^, for example, report prevalence rates for suicidal ideation that ranged from 4.1% in Indonesia to 32.1% in Chile whilst the overall cross-national prevalence was 16.2% in females and 12.2% in males. With reference to the UAE specifically, the location of the present study, the prevalence of ideation was marginally higher than the average—14% and 14.4% for females and males, respectively^9^. In the multi-country study conducted by Felez-Nobrega et al.^10^, a wide range for suicide attempts was evident, 3.4% in Indonesia to 67.8% in Samoa, although this latter figure was an outlier. The next nearest figure at the top end of the range was 34.3% in the Soloman Islands, while in the UAE specifically, figures of 12.2% and 12.5% were obtained for suicide attempts amongst males and females, respectively^10^. Elsewhere, comparatively lower rates have been reported by single country studies—13.6% and 10.3% for ideation and attempts, respectively, in Nepal^11^ and 23.2% and 28.3% for ideation and attempts, respectively, in Benin^12^.

Moreover, despite the substantial variation in the prevalence of suicidal behaviour, there appears to be greater consistency across nations with regard to the associated risk factors. Literature has consistently shown that anxiety difficulties, loneliness, bullying victimization, alcohol and drug use, food insecurity, sedentary behaviour, truancy, and poor parental support and monitoring are reliably associated with an increased risk for engagement in suicidal behaviour^9–14^.

Despite the immense prevalence of suicide and the clear and demonstrable impact it poses to the health and wellbeing of young people, the issue remains unexamined in particular parts of the world which in turn hinders public health campaigns and mental health prevention efforts. In the Middle East, for example, mental health research is generally scant in comparison to North American and European settings. Moreover, the study of suicidal behaviour, in particular, is hindered by the prevailing cultural and religious discourse which often views suicide as a shameful act perpetrated by a weak individual and, in a cultural milieu in which family honour is paramount, the individual may be accused of bringing shame upon their family’s name^15^. Additionally, suicide may be viewed as a sinful act or the result of a weak character and a lack of religious devotion^16^. This prevailing conceptualization of suicide has directly impacted the societal response that typically follows—individuals experiencing immense psychological distress may elect to seek or be directed by others to enlist the assistance of spiritual healers rather than the services of mental health professionals^17^.

A further potential impediment to the conduct of research on suicidal behaviour is the fact that suicide is still regarded as a criminal offence in a number of countries^18^. In the UAE, until very recently, those found to be guilty of the ‘crime’ could be punished with a minimum sentence of 6 months and/ or a 5000 dirham fine (equivalent to approximately 1400 US dollars). In November 2020, the UAE repealed this law effectively decriminalizing suicide and promoting a clear message that laws that seek to punish those afflicted by psychological distress are counter-therapeutic and that rather than punishment such individuals require care, support, and assistance. Despite these apparent macro level changes to the conceptualization of mental health difficulties and suicidal behaviour in this part of the Middle East, at a micro, community, and family-level, it is reported that individuals who experience mental health difficulties continue to experience overt stigma. Moreover, the literature suggests that this stigma that originates in societal structures tends to be internalised, is directed at the self, and thus serves to further compound the individual’s mental health struggles^17^.

Given the nuances of this cultural milieu, the conduct of research examining suicidality is often difficult in Middle Eastern settings. Researchers may experience difficulty recruiting participants or indeed obtaining the ethical approvals required to conduct such research. Thus, as a contribution to the literature, we employed the use of data from the Global School-based Student Health Survey, a readily available, nationally representative dataset to examine the prevalence of suicidal behaviour among school-going adolescents in the UAE. We also investigated whether a range of psychosocial, risky health, and socio-environmental factors would contribute to elevating the risk for engagement in suicidal behaviour.

### The UAE sociocultural context

The population of the UAE is characterized by its diverse cultural landscape, deeply rooted religious traditions, and evolving perspectives on mental health. Emirati society, as a whole, reflects these characteristics. Culturally, the UAE is a melting pot of nationalities, with a significant expatriate population contributing to its multicultural fabric. However, at its core, Emirati culture is influenced by Arab traditions, Bedouin heritage, and a strong sense of hospitality. Religion plays a central role in the lives of Emiratis, with Islam being the predominant faith. Islamic values and practices shape various aspects of society, influencing social norms and behaviors. Concerning mental health, there has been a growing recognition of the importance of addressing mental well-being in recent years. While there may still be stigma associated with mental health issues, Emirati society is increasingly open to discussions about mental health, with religious leaders and community figures playing a role in raising awareness and promoting understanding. Embracing both tradition and modernity, the UAE continues to navigate the intersection of culture, religion, and evolving attitudes toward mental health in its diverse and dynamic society.

## Materials and method

### Sample design

We extracted publicly available data for this study’s analyses from the Global School-based Student Health Survey (GSHS), a survey designed to collect data from school-going adolescents across a number of varying countries. Further details about the survey’s design and content can be accessed at https://www.who.int/teams/noncommunicable-diseases/surveillance/data/united-arab-emirates and http://www.cdc.gov/gshs. The GSHS initiative was developed and managed by an alliance of the WHO, the US Centers for Disease Control and Prevention, and the United Nations.

The principal goal of the GSHS is to examine young people’s health behaviour and measure the range of risk and protective factors associated with morbidity and mortality in representative national samples of school-going adolescents^19^. We specifically extracted data collected in the UAE during the 2016 data collection period. A standardized 2-stage probability sampling design was employed to select schools to be sampled. In stage one, schools were selected with probability proportional to size sampling. Stage two involved the random selection of classrooms that included students aged 11 to 18 years in each selected school. All students in the identified classrooms were eligible to participate. Surveys were administered during regular class time and in the local language (this would be primarily Arabic in some parts of the UAE but typically English is also spoken as a first language in urban areas). Questions on the survey were multiple-choice and completed questionnaires could be electronically scanned to record the results. The GSHS was approved for conduct in the UAE by the government’s Ministry of Health. Participation in the GSHS was entirely voluntary and anonymous. Informed consent was obtained from the students, their parents, and/or the respective school officials where appropriate. Data were weighted for nonresponse and probability selection.

### Outcome variable

The GSHS measured three aspects of suicidal behaviour—suicidal ideation was measured using the item, “during the past 12 months, did you ever seriously consider attempting suicide?”; the presence of a suicide plan was captured using the item, “during the past 12 months, did you make a plan about how you would attempt suicide?”; and suicide attempts was measured using the item, “during the past 12 months, how many times did you actually attempt suicide?”. The items measuring ideation and plan provided binary response options (yes or no) while the attempts item provided the following possible response options: “0”, “1”, “2 or 3”, “4 or 5”, and “6 or more” times. For this item, participant responses were recoded from the original database as a binary item that reflected either no attempts (rated as 0) or the presence of 1 or more attempts (rated as 1). Data for these three questions were combined to create the suicidal behaviour outcome variable—students were recorded as having reported affirmative responses to any of the three measured sub-behaviours (ideation, presence of a plan, or at least one attempt) or not.

### Predictor variables

We examined the association of a number of demographic, psychosocial, risky health, and socio-environmental factors with suicidal behaviour. These variables have previously been subjected to similar analyses in earlier reports of GSHS results and in similar examinations of the survey’s data in other regions of the world^1,10,11,20–22^. We would therefore be able to compare the results of our analyses with this preceding literature. These variables and their associated coding schemes are illustrated in Table 1—variables are classified according to their relevant variable type (i.e., demographic, psychosocial, risky health, or socio-environmental).

**Table 1:** Complete list of independent variables.

Variable	Variable coding	Variable type
Age	11–18 years (coded categorically)	Demographic variables
Gender	1 = Male	
	2 = Female	
School grade	1 = GR7	
	2 = GR8	
	3 = GR9	
	4 = GR10	
	5 = GR11	
	6 = GR12	
Suicidal ideation	0 = No	Psychosocial factors
	1 = Yes	
Suicide plan	0 = No	
	1 = Yes	
Suicide attempts	0 = No	
	1 = Yes	
Loneliness^a^	0, No = never/rarely/sometimes	
	1, Yes = most of the time/always	
Anxiety^a^	0, No = never/rarely/sometimes	
	1, Yes = most of time/always	
Bullied^a^	0, No = never	
	1, Yes = one or more days	
Cigarette Smoking^b^	0 = No	Risky health behaviors
	1 = Yes	
Other tobacco^b^	0 = No	
	1 = Yes	
Homework check^b^	0, No = never/rarely/sometimes	Socio-environmental factors
	1, Yes = most of the time/always	
Parent understanding^b^	0, No = never/rarely/sometimes	
	1, Yes = most of the time/always	
Parent monitor free time^b^	0, No = never/rarely/sometimes	
	1, Yes = most of the time/always	
Sleep difficulties	0 = No	
	1 = Yes	
Physical violence^a^	0 = No	
	1 = Yes	
Food insecurity	0, No = never	
	1, Yes = rarely/sometimes/most of the time/always	

^a^In the past 12 months; ^b^In the past 30 days.

### Multiple adverse experience variable

The procedure previously employed in Khan et al.^20^ was followed in computing a variable representative of each individual’s multiple adverse experiences (MAE). In generating each participant’s MAE score, adverse experiences were first classified into one of three sub-categories (psychosocial, risky health or socio-environmental) and then a score of 1 was assigned to each adverse experience in each of the three categories (the experiences comprising these three sub-categories are illustrated in Table 1). Thus, for example, if an individual participant reported having experienced three adverse experiences in each of the three sub-categories, their MAE score for each category would be 3 but the overall MAE score would total 9.

### Statistical analysis

The age-adjusted prevalence of suicidal behaviour in this sample was calculated using data originating from the WHO relating to the age distribution of suicidal behaviour in the UAE. A generalized estimating equation-modified Poison regression approach with robust error variance was employed to examine the associations between young people’s suicidal behaviour and a range of psychosocial, risky health, and socio-environmental factors, associations that were expressed using of risk ratios (RRs). Both unadjusted and adjusted models were produced for the composite binary outcome variable (ideation + presence of a plan + attempts = suicidal behaviour) with individual psychosocial, risky health, and socio-environmental factors inserted as potential predictors. Similar analyses were conducted for the multiple adverse experience variable. RRs were computed to represent the magnitude of the association and associated 95% confidence intervals were produced for significance testing. Significance was set at *p* < 0.05.

We also separately examined the impact of psychosocial, risky health, and socio-environmental factors on each of suicidal ideation, the presence of a suicide plan, and suicide attempts. The complexity of the survey’s design and sampling weights were considered in the completion of all analyses. All analyses were conducted using STATA Version 13.0^23^.

### Ethics approval and consent to participate

The GSHS was approved for conduct in the UAE by the government’s Ministry of Health. Participation in the GSHS was entirely voluntary and anonymous. Informed consent was obtained from the students, their parents, and/or the respective school officials where appropriate. All methods employed in this study were conducted in accordance with the Declaration of Helsinki and its later amendments.

## Results

### Sample characteristics and prevalence of suicidal behaviour

Our analyses included data from a total of 5826 participants. The sample consisted of relatively equivalent numbers of girls and boys (52.4% versus 47.6%, respectively) and a relatively equivalent distribution was evident across the varying age spectrum with the majority of participants falling within the 13 to 16 age range. Table 2 depicts the sample’s characteristics and illustrates both the unadjusted and age-adjusted prevalence of suicidal behaviour in relation to these sample characteristics. Approximately a third of the sample (35.5%) reported the experience of at least one adverse psychosocial risk factor. Relatively equivalent proportions reported the experience of a single risk factor versus the presence of two factors (proportions of 16.6% versus 17.3%, respectively). A total of 26% reported having been bullied, 16.7% struggled with anxiety, and 14.2% endured loneliness. Moreover, approximately half of the sample reported the presence of at least one risky health behaviour (53.6%) while the vast majority of participants, a total of 83.1%, reported the experience of at least one adverse socio-environmental risk factor.

**Table 2:** Age-adjusted prevalence of suicidal behaviour according to different socio-demographic and adolescent’s adverse experiences.

Variables	Total		Prevalence of suicidal behavior			
			Unadjusted		Age-adjusted	
	%	95% CI	%	95% CI	%	95% CI
Suicidal behavior	57	(65.1–68.3)	55	(52.9–67.0)	54	(53.7–64.6)
Age						
11	0.7	(− 1.26 to 2.66)	0.2	(− 1.76 to 2.16)	0.2	(− 1.76 to 2.16)
12	4.8	(3.4–6.6)	1.6	(1.2–3.8)	1.6	(1.3–3.7)
13	15.6	(12.7–18.2)	5.2	(5.1–14.1)	4.8	(2.4–9.4)
14	19.3	(9.9–19.5)	6.4	(6.2–12.9)	6.3	(5.5–12.9)
15	19.8	(7.7–19.9)	6.6	(6.3–12.4)	6.6	(6.3–8.6)
16	18.7	(9.5–19.9)	6.8	(6.5–8.4)	5.9	(5.4–12.9)
17	14.9	(13.2–17.1)	5.0	(3.2–12.4)	4.9	(2.4–11.0)
18	6.1	(5.7–9.6)	2.0	(1.9–5.6)	1.9	(1.7–9.6)
Gender						
Male	47.6	(42.3–65.0)	15.8	(14.3–18.0)	14.2	(9.2–14.8)
Female	52.4	(49.4–53.9)	17.2	(12.6–17.4)	16.8	(12.7–18.9)
School grade						
GR7	4.3	(4.0–5.3)	1.4	(1.3–2.0)	1.6	(1.5–4.6)
GR8	21.2	(19.4–22.9)	7.0	(6.1–12.5)	8.0	(4.5–12.1)
GR9	20.2	(16.0–25.3)	6.6	(5.3–11.2)	5.6	(5.2–14.2)
GR10	21.1	(20.9–29.2)	6.9	(6.2–13.2)	7.4	(6.3–11.5)
GR11	16.7	(15.8–17.0)	7.6	(4.6–9.7)	5.6	(5.2–14.4)
GR12	16.3	(15.4- 22.9)	5.4	(4.4–11.9)	5.4	(4.6–10.3)
Psychosocial factors						
Loneliness						
No	85.8	(82.3–92.6)	25.7	(12.2–29.3)	28.5	(27.2–30.6)
Yes	14.2	(12.4–17.7)	4.7	(3.5–11.2)	4.9	(4.7–12.2)
Anxiety						
No	83.3	(81.0–90.6)	27.5	(22.1–32.3)	25.5	(24.4–32.4)
Yes	16.7	(12.1–19.2)	7.9	(6.5–9.2)	5.5	(3.5–12.2)
Bullied						
No	74.0	(81.0–90.6)	23.4	(18.4–27.6)	23.4	(22.4–27.6)
Yes	26.0	(12.1–29.2)	8.2	(7.2–12.1)	8.2	(7.5–13.1)
No. of psychosocial factors						
0	64.5	(61.2–67.8)	21.7	(17.2–23.4)	20.4	(18.6–27.8)
1	16.6	(11.9–17.5)	18.2	(11.7–19.1)	17.2	(13.4–18.9)
2	17.3	(12.5–18.2)	20.4	(18.2–21.5)	22.4	(12.5–28.2)
≥ 3	1.6	(1.2–9.2)	8.2	(7.2–19.2)	18.2	(9.2–21.2)
Risky health factors						
Cigarette smoking						
No	88.1	(81.0–93.6)	28.5	(26.5–30.6)	27.5	(25.7–30.3)
Yes	11.9	(9.1–15.2)	3.8	(2.1–8.8)	4.8	(2.7–5.2)
Other tobacco						
No	85.7	(82.7–90.9)	28.4	(26.5–31.2)	27.4	(22.3–29.4)
Yes	14.3	(11.3–16.2)	6.7	(3.5–9.8)	4.7	(2.5–7.4)
Physical violence						
No	61.6	(55.7–67.8)	22.5	(21.2–28.8)	20.5	(18.5–24.2)
Yes	38.4	(33.3–43.6)	12.8	(9.7–16.8)	14.2	(13.5–18.3)
No. of risky health behaviors						
0	45.9	(41.6–67.8)	21.9	(17.2–22.7)	20.4	(18.6–27.8)
1	30.6	(21.8–37.9)	16.8	(13.2–18.4)	17.2	(13.4–18.9)
2	20.3	(12.5–25.2)	32.4	(21.5–38.7)	28.2	(12.5–34.5)
≥ 3	2.7	(1.2–9.2)	18.2	(17.2–24.2)	17.4	(9.3–21.2)
Socio-environmental factors						
Homework check						
No	21.6	(18.4–23.8)	7.0	(5.5–11.7)	7.6	(4.1–15.0)
Yes	78.4	(73.5–89.8)	25.6	(23.6–29.2)	23.6	(19.2–25.2)
Parent understanding						
No	20.3	(16.1–24.3)	6.3	(4.2–9.7)	6.6	(5.9–11.8)
Yes	79.7	(75.1–90.2)	22.8	(20.2–26.9)	25.8	(19.2–30.9)
Parent monitor free time						
No	15.9	(10.4–18.5)	5.2	(3.9–8.7)	5.8	(3.8–10.9)
Yes	84.1	(79.9–90.0)	26.4	(22.7–30.9)	27.4	(22.2–36.4)
Food insecurity						
No	51.2	(45.2–66.4)	17.4	(14.2–29.2)	18.1	(15.7–29.5)
Yes	48.8	(43.6–56.7)	15.2	(9.2–27.3)	16.9	(11.2–26.9)
No. of socio-environmental factors						
0	16.7	(21.6–19.8)	8.7	(4.2–9.9)	10.4	(8.6–12.2)
1	25.8	(20.5–26.9)	16.8	(13.2–18.4)	17.2	(15.1–18.4)
2	22.7	(20.5–25.6)	12.4	(10.5–15.7)	14.5	(12.5–19.8)
≥ 3	34.6	(31.7–36.2)	20.2	(19.9–24.2)	21.4	(17.2–22.3)

All percentages are weighted. Percentages may not be total 100% because of rounding. ^a^In the past 12 months; ^b^In the past 30 days.

**Figure 1:**
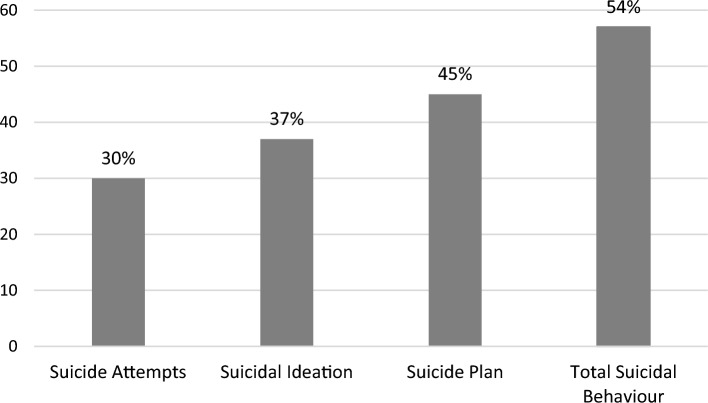
The prevalence of suicide attempts, suicidal ideation, and the presence of a suicide plan amongst the sample of adolescents in the UAE. Data are expressed as percentages.

The unadjusted prevalence of suicidal behaviour in the total sample was 55% while the overall age-adjusted prevalence was 54%. Specifically, 37% reported suicidal ideation, 45% had formulated a suicide plan, and 30% had attempted suicide during the preceding 12 months (see Fig. 1). A number of psychosocial factors appeared to be related to an increased prevalence of suicidal behaviour. Specifically, when considering the age-adjusted prevalence of suicidal behaviour, the self-reported prevalence of suicidal behaviour was higher amongst adolescents who had experienced anxiety difficulties compared to those who had not (25.5% versus 5.5%, respectively), those who had been the victims of bullying compared to those who had not (23.4% versus 8.2%, respectively), and among adolescents who reported experiencing loneliness compared to those who had not (28.5% versus 4.9%, respectively). The age-adjusted prevalence was highest among adolescents reported having experienced at least 2 concurrent psychosocial risk factors (22.4%).

When considering the contribution of the risky health factors, age-adjusted prevalence was higher amongst those who experienced physical violence (20.5% versus 14.2%), those who smoked cigarettes (27.5% versus 4.8%) as well as other forms of tobacco (27.4% versus 4.7%). Age-adjusted prevalence was also significantly higher among adolescents whose parents rarely checked their homework (23.6% versus 7.6%), whose parents failed to monitor their children’s free time (27.4% versus 5.8%), and those who reported a low level of understanding between themselves and their parents (25.8% versus 6.6%). Individuals who reported the presence of at least 3 concurrent adverse socio-environmental risk factors also reported the highest prevalence of suicidal behaviour (21.4%).

### Risk factors associated with suicidal behaviour

Table 3 illustrates the results of analyses demonstrating the associations of the variously examined risk factors with suicidal behaviour. Risk for suicidal behaviour was significantly elevated amongst individuals who experienced anxiety (ARR 1.17; 95% CI 1.12–1.23) and those who reported loneliness (ARR 1.19; 95% CI 1.13–1.25). Examination of individuals who experienced being bullied did not produce a statistically significant association (ARR 1.14; 95% CI 1.09–1.18). Additionally, those who smoked cigarettes were also at heightened risk for suicidal behaviour (ARR 1.32; 95% CI 1.22–1.42). All of the socio-environmental risk factors examined produced non-significant associations.

**Table 3:** Association between socio-demographics and adolescent’s adverse experiences and suicidal behaviour.

Risk factor	Prevalence of suicidal behavior			
	URR	95% CI	ARR^a^	95% CI
Age (years)				
11–12^(RC)^	1.00	–	1.00	–
13	0.327	(0.13–0.77)	0.345	(0.15–0.78)
14	0.523	(0.33–0.80)	0.423	(0.29–0.75)
15	0.767	(0.52–1.12)	0.762	(0.49–0.999)
16	0.706	(0.48–1.03)	0.602	(0.46–0.998)
17	0.688	(0.46–1.01)	0.670	(0.41–0.995)
18	0.688	(0.46–1.02)	0.675	(0.32–0.982)
Gender				
Male^(RC)^	1.00	–	1.00	–
Female	0.97	(0.78–1.20)	0.99	(0.97–1.02)
School grade				
GR7^(RC)^	1.00	–	1.00	–
GR8	2.00	(0.73–5.50)	1.92	(0.69–4.50)
GR9	1.12	(0.81–1.53)	1.02	(0.75–1.40)
GR10	1.07	(0.77–1.48)	1.02	(0.72–1.35)
GR11	1.00	(0.72–1.38)	0.989	(0.65–1.25)
GR12	0.918	(0.64–1.31)	0.818	(0.55–1.22)
Psychosocial factors				
Loneliness				
No^(RC)^	1.00	–	1.00	–
Yes	3.11^d^	(2.43–3.97)	1.19^c^	(1.13–1.25)
Anxiety				
No^(RC)^	1.00	–	1.00	–
Yes	2.97^d^	(2.34–3.78)	1.17^c^	(1.12–1.23)
Bullied				
No^(RC)^	1.00	–	1.00	–
Yes	2.72^d^	(2.16–3.43)	1.14	(1.09–1.18)
Health risk factors				
Cigarette smoking				
No^(RC)^	1.00	–	1.00	–
Yes	4.59^d^	(3.52–5.99)	1.32^b^	(1.22–1.42)
Other tobacco				
No^(RC)^	1.00	–	1.00	–
Yes	3.42^d^	(2.67–4.39)	1.22	(1.15–1.29)
Sleep difficulties				
No^(RC)^	1.00	–	1.00	–
Yes	2.11^d^	(1.62–2.75)	1.07	(1.05–1.100)
Physical violence				
No^(RC)^	1.00	–	1.00	–
Yes	2.27^d^	(1.83–2.81)	1.10	(1.07–1.13)
Socio-environmental factors				
Homework check				
No^(RC)^	1.00	–	1.00	–
Yes	0.68	(0.53–0.88)	0.95	(0.92–0.98)
Parent understanding				
No^(RC)^	1.00	–	1.00	–
Yes	0.43	(0.34–0.55)	0.89	(0.85–0.92)
Parent monitor free time				
No^(RC)^	1.00	–	1.00	–
Yes	0.43	(0.33–0.56)	0.88	(0.84–0.92)
Food insecurity				
No^(RC)^	1.00	–	1.00	–
Yes	1.53^b^	(1.23–1.89)	1.04	(1.02–1.07)

^RC^Reference category, URR (unadjusted risk ratio), CI (confidence interval), ARR (adjusted risk ratio). ^a^Model was adjusted for all the predictors included in this table. Values with superscript b, c, and d indicate *p* < 0.05, *p* < 0.01, and *p* < 0.001, respectively.

### Multiple adverse experiences

Table 4 illustrates the effect of MAEs in relation to adolescents’ suicidal behaviour. The results indicate that, when age, school grade, and gender were controlled for, in comparison to adolescents who did not report any adverse experiences, those who reported the experience of 1, 2, and ≥ 3 adverse psychosocial experiences exhibited elevated risk for suicidal behaviour—risk ratios of 1.26 (95% CI 1.32–2.50), 1.36 (95% CI 1.38–2.62), and 1.30 (95% CI 1.45–2.77), respectively. Our analyses also indicated that those who reported the presence of 1 and ≥ 2 risky health factors were similarly at increased likelihood of exhibiting suicidal behaviour, adjusted risk ratios of 1.12 (95% CI 1.02–2.26) and 2.14 (95% CI 1.99–4.88), respectively. In relation to adverse socio-environmental factors, adolescents who reported the experience of 2 and ≥ 3 factors were at elevated risk for suicidal behaviour, adjusted risk ratios of 1.32 (95% CI 1.65–2.99) and 1.57 (95% CI 1.87–3.01), respectively.

**Table 4:** Effect of multiple adverse experiences on adolescents’ suicidal behaviour.

Multiple adverse experiences	Suicidal behavior			
	URR	95% CI	ARR^a^	95% CI
No. of psychosocial factors				
0^(RC)^	1.00	–	1.00	–
1	1.93^d^	(1.44–2.61)	1.26^c^	(1.32–2.50)
2	2.11^d^	(1.58–2.82)	1.36^c^	(1.38–2.62)
≥ 3	2.01^d^	(1.56–2.59)	1.30^c^	(1.45–2.77)
No. of risky-health behaviors				
0^(RC)^	1.00	–	1.00	–
1	1.59^c^	(1.07–2.36)	1.12^c^	(1.02–2.26)
≥ 2	3.35^d^	(2.23–5.04)	2.14^d^	(1.99–4.88)
No. of adverse socio-environmental factors				
0^(RC)^	1.00	–	1.00	–
1	1.15^c^	(.912–1.45)	1.00	(0.832–1.12)
2	2.04^d^	(1.52–2.73)	1.32^c^	(1.43–2.62)
≥ 3	2.49^d^	(1.95–3.19)	1.57^b^	(1.45–2.99)

^RC^reference category, URR (unadjusted risk ratio), CI (confidence interval), ARR (adjusted risk ratio). ^a^Model was adjusted for all the predictors included in this table. Values with superscript b, c, and d indicate *p* < 0.05, *p* < 0.01, and *p* < 0.001, respectively.

Additionally, we also tested the association of MAEs in relation to each of the three specific dimensions of suicidal behaviour assessed—namely, suicidal ideation, suicide plan, and suicide attempts. Table 5 illustrates the adjusted risk ratios, all of which were statistically significant, and suggestive of a dose-dependent relationship between multiple, accruing, adverse experiences and suicidal behaviour. Specifically, the adjusted risk ratios indicative of the association between increasing numbers of concurrent MAEs and risk for suicidal behaviour increased, in some cases, exponentially, as the number MAEs increased. This was true for each of suicidal ideation, suicide plan, and suicide attempts as well as for each of the three types of risk factors assessed (i.e., psychosocial, risky health, and socio-environmental).

**Table 5:** Effect of multiple adverse experiences on adolescents’ suicidal behaviour.

Multiple adverse experiences	Suicidal ideation ARR (95% CI)	Suicidal plan ARR (95% CI)	Suicidal attempt ARR (95% CI)
No. of psychosocial factors			
0^(RC)^	1.00	1.00	1.00
1	2.85 (2.31–3.51)^d^	2.37 (1.88–2.99)^d^	2.72 (2.16–4.34)
2	4.22 (3.40–5.23)^d^	3.03 (2.38–3.87)^d^	2.97 (2.34–3.78)^d^
≥ 3	4.53 (3.64–5.65)^d^	3.24 (2.56–4.09)^d^	3.11 (2.43–3.97)^d^
No. of risky health behaviors			
0^(RC)^	1.00	1.00	1.00
1	3.03 (2.40–3.82)^d^	2.31 (1.78–3.00)^d^	3.42 (2.67–4.39)^d^
≥ 2	4.08 (3.17–5.24)^d^	3.16 (2.40–4.17)^d^	4.59 (3.52–5.95)^d^
No. of adverse socio-environmental factors			
0^(RC)^	1.00	1.00	1.00
1	1.78 (1.46–2.16)^c^	1.59 (1.29–1.97)^b^	1.53 (1.23–1.89)^c^
2	1.94 (1.59–2.35)^b^	1.99 (1.61–2.46)^d^	2.11 (1.62–2.75)^b^
≥ 3	2.48 (1.94–3.16)^d^	2.02 (1.56–2.61)^d^	2.27 (1.83–2.81)^d^

^RC^Reference category, URR (unadjusted risk ratio), CI (confidence interval), ARR (adjusted risk ratio). Values with superscript b, c, and d indicate *p* < 0.05, *p* < 0.01, and *p* < 0.001, respectively.

## Discussion

This study employed a nationally representative dataset to examine the prevalence of suicidal ideation, the formulation of a suicide plan, and suicide attempts amongst a sample of school-going adolescents resident in the UAE. We also computed the magnitude of risk of a number of variables potentially associated with an elevated likelihood of engagement in suicidal behaviour. In this sample, the age-adjusted prevalence of suicidal behaviour—encompassing the three sub-elements of suicidal ideation, suicide plan, and suicide attempts—was 54%, a figure that is high in comparison to the majority of adolescents typically sampled in the GSHS studies. Numerous risk factors were determined to be related to an elevated risk for suicidal behaviour in this sample. These risk factors encompassed psychosocial, risky health, as well as socio-environmental factors. Moreover, our results also suggest that the cumulative effect of multiple adverse experiences (or risk factors) serve to further multiply the risk for suicidal behaviour in this particular population.

When comparing our computed prevalence of suicidal behaviour using the 2016 GSHS sample from the UAE to that of other studies as well as with previous versions of the GSHS, our obtained prevalence rates are high. Our computed prevalence rates are comparable to the upper end of the reported ranges in previous multi-national studies but substantially higher than most of the included countries in these studies^9,10^. This is the case both for the overall rate of suicidal behaviour but also for each of the three component behaviours. Specifically, the obtained overall prevalence of ideation (37%) was only comparable to Chile (32.1%) in McKinnon et al.’s^9^ analysis. In relation to suicide attempts, only three of the 48 included countries in Felez-Nobrega et al.’s^10^ synthesis was comparable to this study’s prevalence rate of 30%. Our computed results are also higher than those reported in single-country studies from Benin^12^, Bangladesh^20^, and Nepal^11^.

These results are surprising for two reasons. First, these figures are comparable to that of developing countries where rates of suicidality typically tend to be higher in comparison to high-income countries such as the UAE^24,25^. Second, our obtained results are also higher than those reported by previous rounds of GSHS data collection in the UAE. This may suggest that rates of suicidal behaviour may indeed be rising in this country and, if so, further exploration of this phenomenon’s catalysts should be investigated. Moreover, perhaps, given the recent decriminalization of suicide in this country, responses to previous versions of the study may have misrepresented the true extent of the issue as individuals may have underreported their behaviours fearing legal reprisal. Given that individuals can now freely express their struggles with suicidality without the threat of legal repercussions, one might potentially expect reporting to increase and so too the documented prevalence rates.

Our analyses also suggest that a number of psychosocial factors are associated with an increased risk for engagement in suicidal behaviour and this concurs with previous literature^11,26–28^. With regard to psychosocial factors, both anxiety and loneliness emerged as being significantly associated with suicidal behaviour. Indeed, a plethora of research, both cross-sectional and longitudinal, particularly in the adult population, have demonstrated an association between the presence of anxiety difficulties and suicidal ideation and attempts^29–31^. The literature suggests that comorbid psychopathologies, such as personality disordered symptomology and the presence of a primary mood disorder, elevates the magnitude of this association^29–31^. Our results suggest that a similar association amongst adolescents is present but the direction of causality of this relationship should be tested in future investigations. Moreover, research also demonstrates that the experience of loneliness is associated with both mood and anxiety symptoms which, in turn, are related to a higher risk for suicidal behaviour^32^. Given that peer relationships and friendships serve as an important supportive mechanism for most people during times of emotional distress, for adolescents who experience psychosocial distress such as depression or anxiety difficulties but also concurrently have few close relationships or friendships, the magnitude of their distress is likely to be exacerbated. Indeed, evidence suggests that peer relationships for adolescents are an important vehicle for motivation, support, inspiration, and modelling of healthy behaviour—for example, the development of prosocial coping skills, feedback of healthy behaviours, and the provision of access to adaptive cognitive styles^33^.

The only risky health behaviour that appeared to be associated with suicidal behaviour was tobacco smoking. Smoking has previously been shown, both in the context of large-scale cross-sectional and longitudinal research to be related to suicidality, particularly current daily smoking as opposed to a previous history of smoking^34,35^. Biological explanations have been proposed that attempt to explain this demonstrated association, such as evidence of lowered monoamine oxidase activity present in current smokers but absent in ex-smokers—this, in turn, may play a central role in central nervous system serotonin metabolism but the proposition remains unclear^36^. Additionally, this demonstrated association may be the result of individuals with suicidal ideation being less concerned with the long-term negative health implications of tobacco consumption. Thus, smoking behaviour may therefore be a potential modifiable risk factor that should be targeted, perhaps in the form of smoking cessation interventions delivered in school settings. Doing so successfully could potentially reduce the proportion of risk it contributes towards suicidal behaviour.

Whilst none of the socio-environmental factors emerged as significant, it is interesting to note that, when proportional differences in suicidal behaviour were examined using the risk factor as the stratification variable (Table 2), suicidal behaviour occurred more frequently amongst adolescents whose parents rarely checked their homework, and failed to monitor their children’s free time, and those who reported a low level of understanding between themselves and their parents. These results highlight the integral role played by the parent–child relationship and the need for adolescents to have a healthy connection to a supportive familial unit, both of which serve as protective mechanisms and promote healthy psycho-emotional functioning. These protective functions and conversely, the detrimental effect of the lack thereof, have previously been demonstrated elsewhere^37^. Parent–child interventions designed to promote healthy relationships and parenting skills may represent a potentially modifiable risk factor for suicidal behaviour in this locale.

### Limitations

Despite its important contribution to the literature, this study possesses a number of limitations, which should be borne in mind when reflecting upon its results. While we have been able to identify, using a cross-sectional methodology, that a number of risk factors are associated with suicidal behaviour, we are unable to draw any conclusions about the nature and direction of their causality. Cross-sectional studies of this type are important in determining which factors may be especially relevant to a given outcome (in this case, suicidal behaviour) but studies with longitudinal designs in which measurements are taken from participants at multiple timepoints would more reliably facilitate an understanding of the causal relationship at play between these variables. Doing so, however, at the scale employed by the GSHS would be a substantial undertaking that would require meticulous planning and monitoring to ensure fidelity.

A second issue that places a limitation on the current study is our use of an existing publicly available dataset. Given that we did not design the study and the survey ourselves, the risk factors included in our analyses were limited to those that were available. There may therefore be a variety of additional risk factors that indeed also be relevant in increasing risk for suicidal behaviour but were not included in these analyses.

Finally, the GSHS is a self-report measure that is completed by school-going adolescents in the identified classrooms. This poses two additional limitations. First, the responses to self-report measures may be prone to social desirability or susceptible to recall bias^38^. Second, these results are reflective of the adolescents who attend school, there may be some who do not, and those who attended on the day of data collection. Therefore, there remains the possibility that the content of the GSHS may not necessarily be representative of all adolescents in this locale.

### Implications

This study’s principal strengths are its use of a large, nationally representative sample which increases the study’s power and therefore the reliability of its results. Its use of a standardized survey across all enrolled nations is an additional strength as it enables cross-national comparisons to be made. But most importantly, our findings draw attention to what appears to be a significant mental health issue, that of suicidal behaviour amongst school-going adolescents in the UAE. Successful tackling of the issue may only be possible when targeted at multiple points of potential remediation.

From a societal vantage point, these findings necessitate urgent and multi-pronged interventions. The alarming rate of suicidality among adolescents should not be viewed in isolation but rather as potentially indicative of pervasive issues that may span familial, educational, and cultural dimensions. Social norms that stigmatize mental health difficulties must be urgently revised. Public health campaigns that aim to educate and alter pre-existing prejudices could be beneficial in this regard and should be widely promoted via religious entities, welfare organizations, governmental institutions, and in the form of public mental health campaigns^39^. Moreover, mental health policies are needed that are sensitive to the enormity of mental difficulties in this population and enable access to evidence-based psychological support and care. Health providers, ranging from general practitioners to specialized mental health experts, must be attuned to these epidemiological tendencies. Routine screenings for suicidal ideation and associated risk factors should be integrated into primary and secondary care settings, particularly in the context of adolescent health consultations. Healthcare providers should also be equipped with resources, both pharmacological and psychotherapeutic, to address emergent cases effectively.

Given the profound influence that religious institutions hold within the UAE, religious leaders can play a pivotal role in destigmatizing mental health issues. They could serve as a means from which to disseminate messages that are aligned with the core principles of empathy, compassion, and understanding. Leveraging this source of authority to emphasize the importance of mental well-being could be a highly effective approach in a society where religious tenets are deeply ingrained^17^.

Schools are frontline settings where changes in adolescent behaviour are most readily observed. Teachers, thus, bear a significant responsibility for the early identification and referral of at-risk students. Furthermore, curricular interventions that focus on building emotional intelligence, resilience, and adaptive coping strategies should be instated. Peer mentorship programs may serve as an additional layer of support and should be empirically evaluated for their efficacy.

Culturally sensitive approaches to psychotherapy are not just advisable but imperative in this setting. A nuanced understanding of Emirati culture, customs, and religious beliefs should inform the therapeutic modalities employed. For example, cognitive-behavioural therapy, when adapted to align with cultural norms, has shown promise in treating a range of psychological disorders and could be applied to this demographic^40^.

In sum, multi-faceted approaches are needed to tackle this complex issue. Strategies must not only target the adolescents but also involve parents, teachers, and the community at large. Parental seminars that focus on fostering an emotionally nurturing home environment and recognizing early signs of mental distress could be beneficial. School-based programs should aim to create a culture of openness, encouraging discussions around mental well-being. Universal psychoeducational programs could be employed to inculcate adaptive ways of managing risk factors identified in this study, such as anxiety, experiences of bullying, and substance use. By adopting a holistic, multi-sectoral approach that is underpinned by empirical evidence, there is a greater likelihood of mitigating this alarming trend effectively. The gravity of the situation demands concerted action from all sectors of Emirati society.

## Data Availability

The data used in this study are publicly available at https://extranet.who.int/ncdsmicrodata/index.php/catalog/647/study-description.

## Abbreviations

UAE: United Arab Emirates
WHO: World Health Organization
GSHS: Global School-based Student Health Survey
MAE: Multiple adverse experiences
RRs: Risk ratios

## References

[1] Assarsson R (2018). Gender inequality and adolescent suicide ideation across Africa, Asia, the South Pacific and Latin America—A cross-sectional study based on the Global School Health Survey (GSHS). Glob. Health Action.

[2] Coope C (2014). Suicide and the 2008 economic recession: Who is most at risk? Trends in suicide rates in England and Wales 2001–2011. Soc. Sci. Med..

[3] Doran CM, Kinchin I (2020). Economic and epidemiological impact of youth suicide in countries with the highest human development index. PLoS ONE.

[4] Dos Santos JP, Tavares M, Barros PP (2016). More than just numbers: Suicide rates and the economic cycle in Portugal (1910–2013). SSM Popul. Health.

[5] World Health, O (2014). Preventing Suicide: A Global Imperative.

[6] Boerma T, Mathers CD (2015). The World Health Organization and global health estimates: Improving collaboration and capacity. BMC Med..

[7] Lim KS (2019). Global lifetime and 12-month prevalence of suicidal behavior, deliberate self-harm and non-suicidal self-injury in children and adolescents between 1989 and 2018: A meta-analysis. Int. J. Environ. Res. Public Health.

[8] Liu ZZ (2018). Suicidal behaviours among Chinese adolescents exposed to suicide attempt or death. Epidemiol. Psychiatr. Sci..

[9] McKinnon B (2016). Adolescent suicidal behaviours in 32 low- and middle-income countries. Bull. World Health Organ..

[10] Felez-Nobrega M (2020). Sex difference in the association between physical activity and suicide attempts among adolescents from 48 countries: A global perspective. J. Affect. Disord..

[11] Pandey AR (2019). Factors associated with suicidal ideation and suicidal attempts among adolescent students in Nepal: Findings from Global School-based Students Health Survey. PLoS ONE.

[12] Randall JR (2014). Suicidal behaviour and related risk factors among school-aged youth in the Republic of Benin. PLoS ONE.

[13] Koyanagi A (2019). Bullying victimization and suicide attempt among adolescents aged 12–15 years from 48 countries. J. Am. Acad. Child. Adolesc. Psychiatry.

[14] Vancampfort D (2019). Leisure-time sedentary behavior and suicide attempt among 126,392 adolescents in 43 countries. J. Affect. Disord..

[15] Shah A, Chandia M (2010). The relationship between suicide and Islam: A cross-national study. J. Inj. Violence Res..

[16] Morad M (2005). A review of suicide behavior among Arab adolescents. ScientificWorldJournal.

[17] Vally Z (2018). Public stigma and attitudes toward psychological help-seeking in the United Arab Emirates: The mediational role of self-stigma. Perspect. Psychiatr. Care.

[18] Mishara BL, Weisstub DN (2016). The legal status of suicide: A global review. Int. J. Law Psychiatry.

[19] Johnson RK (2019). The global school-based student health survey as a tool to guide adolescent health interventions in rural Guatemala. BMC Public Health.

[20] Khan MMA (2020). Suicidal behavior among school-going adolescents in Bangladesh: Findings of the global school-based student health survey. Soc. Psychiatry Psychiatr. Epidemiol..

[21] Rahman MM (2020). Bullying victimization and adverse health behaviors among school-going adolescents in South Asia: Findings from the global school-based student health survey. Depress. Anxiety.

[22] Han L (2019). Unintentional injuries and violence among adolescents aged 12–15 years in 68 low-income and middle-income countries: A secondary analysis of data from the Global School-Based Student Health Survey. Lancet Child Adolesc. Health.

[23] *Stata multivariate statistics reference manual : release 10*. (StataCorp LP, 2007) ©2007.

[24] Bantjes J (2016). Poverty and suicide research in low- and middle-income countries: Systematic mapping of literature published in English and a proposed research agenda. Glob. Ment. Health.

[25] Iemmi V (2016). Suicide and poverty in low-income and middle-income countries: A systematic review. Lancet Psychiatry.

[26] Crawford EA (2019). Somatic symptoms of anxiety and suicide ideation among treatment-seeking youth with anxiety disorders. Suicide Life Threat Behav..

[27] Endo K (2017). Preference for solitude, social isolation, suicidal ideation, and self-harm in adolescents. J. Adolesc. Health.

[28] Ibrahim N, Amit N, Suen MW (2014). Psychological factors as predictors of suicidal ideation among adolescents in Malaysia. PLoS ONE.

[29] Teismann T (2018). Suicidal ideation in primary care patients suffering from panic disorder with or without agoraphobia. BMC Psychiatry.

[30] Sareen J (2005). Anxiety disorders and risk for suicidal ideation and suicide attempts: A population-based longitudinal study of adults. Arch Gen. Psychiatry.

[31] Nepon J (2010). The relationship between anxiety disorders and suicide attempts: Findings from the National Epidemiologic Survey on Alcohol and Related Conditions. Depress Anxiety.

[32] Barnabas RV (2020). Community-based antiretroviral therapy versus standard clinic-based services for HIV in South Africa and Uganda (DO ART): A randomised trial. Lancet. Glob. health.

[33] Lodder GMA (2017). Loneliness in early adolescence: Friendship quantity, friendship quality, and dyadic processes. J. Clin. Child Adolesc. Psychol..

[34] Balbuena L, Tempier R (2015). Independent association of chronic smoking and abstinence with suicide. Psychiatr. Serv..

[35] Poorolajal J, Darvishi N (2016). Smoking and suicide: A meta-analysis. PLoS ONE.

[36] Breslau N (2005). Smoking and the risk of suicidal behavior: A prospective study of a community sample. Arch. Gen. Psychiatry.

[37] Murray L (2016). Randomized controlled trial of a book-sharing intervention in a deprived South African community: Effects on carer-infant interactions, and their relation to infant cognitive and socioemotional outcome. J. Child Psychol. Psychiatry.

[38] Vally Z (2021). Celebrity worship in the United Arab Emirates: An examination of its association with problematic internet use, maladaptive daydreaming, and desire for fame. Psychol. Popul. Media.

[39] Corrigan PW, Matthews AK (2003). Stigma and disclosure: Implications for coming out of the closet. J. Ment. Health.

[40] Mir G (2015). Adapted behavioural activation for the treatment of depression in Muslims. J. Affect. Disord..

